# Composite Coatings for Osteoblast Growth Attachment Fabricated by Matrix-Assisted Pulsed Laser Evaporation

**DOI:** 10.3390/polym14142934

**Published:** 2022-07-20

**Authors:** Valentina Grumezescu, Alexandru Mihai Grumezescu, Anton Ficai, Irina Negut, Bogdan Ștefan Vasile, Bianca Gălățeanu, Ariana Hudiță

**Affiliations:** 1 Lasers Department, National Institute for Lasers, Plasma and Radiation Physics, 077125 Magurele, Romania; negut.irina@inflpr.ro; 2 Department of Science and Engineering of Oxide Materials and Nanomaterials, University Politehnica of Bucharest, 060042 Bucharest, Romania; grumezescu@yahoo.com (A.M.G.); anton.ficai@upb.ro (A.F.); bogdan.vasile@upb.ro (B.Ș.V.); 3 Academy of Romanian Scientists, Ilfov No. 3, 50044 Bucharest, Romania; 4 Research Institute of the University of Bucharest—ICUB, University of Bucharest, 050657 Bucharest, Romania; 5 Department of Biochemistry and Molecular Biology, University of Bucharest, 050095 Bucharest, Romania; bianca.galateanu@bio.unibuc.ro (B.G.); arianahudita@yahoo.com (A.H.)

**Keywords:** hydroxyapatite, fibroblast growth factor, chitosan, metallic implants, biocompatibility, matrix-assisted pulsed laser evaporation (MAPLE)

## Abstract

The bioactive and biocompatible properties of hydroxyapatite (HAp) promote the osseointegration process. HAp is widely used in biomedical applications, especially in orthopedics, as well as a coating material for metallic implants. We obtained composite coatings based on HAp, chitosan (CS), and FGF2 by a matrix-assisted pulsed laser evaporation (MAPLE) technique. The coatings were physico-chemically investigated by means of X-ray Diffraction (XRD), Transmission Electron Microscopy (TEM), Infrared Microscopy (IRM), and Scanning Electron Microscopy (SEM). Further, biological investigations were performed. The MAPLE-composite coatings were tested in vitro on the MC3T3-E1 cell line in order to endorse cell attachment and growth without toxic effects and to promote pre-osteoblast differentiation towards the osteogenic lineage. These coatings can be considered suitable for bone tissue engineering applications that lack toxicity and promotes cell adhesion and proliferation while also sustaining the differentiation of pre-osteoblasts towards mature bone cells.

## 1. Introduction

Bone-related defects and diseases from traumatism, tumors, infection, and congenital deformity occur in millions of people, and in some cases, these can be fatal [1,2]. Usually, the repair and treatment of bone defects cannot be performed without intervention; therefore, it continues to be challenging in dental implantology and orthopedics [1]. The graft-based methods, i.e., allografts, autografts, and xenografts, considered standard treatment options in the healthcare practice, are of concern regarding the immunologic reaction, and potential disease transmission [3,4], availability, and donor site morbidity [5]. However, their drawbacks and potential risks have encouraged surgeons and engineers to find new methods to repair bone defects [6,7].

Tissue engineering, a comprehensive multidisciplinary methodology, has provided a unique option for repairing or replacing biological tissues [8]. In tissue engineering, bone regeneration can be facilitated by various approaches such as bone substitutes, scaffolds, and implant coatings, to create a suitable microenvironment in favor of osseointegration. The materials used for bone tissue engineering must possess good bone conductivity and inductivity [9,10].

Surface modifications using coatings represent multipurpose approaches to facilitate the osseointegration of implantable devices [11]. An ideal coating dedicated to hard tissue repair should entail components, structure, and biomechanical properties similar to natural bone, regulate cell adhesion, proliferation, and differentiation, and provide cells with a microenvironment for osteogenic differentiation [12,13]. Surface modification of implantable medical device surfaces with calcium phosphate is a practical option for improving implant integration and the overall biofunctional performance [14].

With the aim to develop functional and performance-enhanced biomaterials and devices for bone tissue therapy, impressive results have been evidenced with respect to non-degradable biopolymers [15,16]. Besides their indisputable biomechanics and biochemical stability, the facile processability and functionality and the tunable composition and microstructure of such biopolymers represent key aspects of being explored for bone-related applications. In addition, the compositional and microstructural characteristics of mineral hydroxyapatite are of great importance for modulating the intricate molecular, cellular, and biochemical processes that occur both within the natural bone tissue and at the bone/implant interface [17]. Thereby, the modification of non-degradable-based biomaterials with hydroxyapatite represents a multifaceted strategy to engineer new platforms and devices, with boosted bioactivity and superior outcomes regarding the restoration, replacement, and regeneration of hard tissue [18,19,20,21].

Due to its physicochemical and microstructural similitude to the inorganic phase of the natural bone, hydroxyapatite (Ca_10_(PO_4_)_6_(OH)_2_) is an inductive reagent for the proliferation of osteoblasts and the growth and differentiation of mesenchymal stem cells [22,23]. Thanks to its non-immunogenic properties, biocompatibility, bioactivity, and bone conductivity, hydroxyapatite has been extensively utilized in diverse coating applications for hard tissue therapy [24,25,26]. Synthetic hydroxyapatite coatings proved to have high performance in sustaining bone growth around the implant [27,28]. Nevertheless, to boost its brittleness and low mechanical strength, hydroxyapatite can be coupled with or incorporated within high-molecular polymers, which further demonstrate better bone-forming ability [29,30].

Biodegradable polymers with adequate mechanical strength and degradation rate are desirable materials for hard tissue therapy [31]. The polymer must also promote the adhesion of osteoblasts to the coating and, finally, create new bone. Biodegradable polymers, such as polylactic acid (PLA) [32], polylactide-co-glycolide (PLGA) [33,34], and chitosan (CS) have been used to develop hydroxyapatite/polymer coatings for bone regeneration. The latter candidate, CS, is a natural-derived polymer and owing to its exceptional biocompatibility, low toxicity, and biodegradability, CS can be used for bone regeneration [35]. CS coatings support cell adhesion and reduce the number of reactive oxygen species in the surrounding environment, leading to osteoblasts accumulation [36]. The combination of CS-coatings and HAp in the same vector stimulates cell growth [37]. HAp/CS biocomposites can induce osteoinduction and osteointegration and promote bone formation in different bone defects [38].

Angiogenic and osteogenic growth factors, as well as the sequential expression of pro-inflammatory proteins, can encourage cell activity, such as the differentiation of osteoprogenitor cells during bone formation [39]. Growth factors attached to the extracellular matrix of the bone have the potential to offer “indications” for bone growth. For instance, in the bone formation stage, fibroblast growth factor-2 (FGF2) regulates the proliferation and differentiation of osteoblasts and osteocyte formation [40,41].

HAp-CS coatings can be obtained by plasma spraying [42], electrophoretic deposition [43,44], spin coating [45], and laser-assisted methods [46]. From these techniques, the matrix-assisted pulsed laser evaporation (MAPLE) method has several advantages over existing techniques, including the production of uniform coatings of different materials and onto various substrates [47,48]. The high experimental versatility of the MAPLE technique allows for the synthesis of delicate compounds (e.g., proteins or polymers) in the form of thin films without altering the stability of their functional characteristics. Specific for MAPLE is the use of a cryogenic target made from the compounds of interest, in our case HAp, CS, and FGF2, diluted in a proper solvent. The laser beam hits the target placed in the vacuumed deposition chamber and transfers the compounds without any damage [41,49].

In our current study, we aimed to obtain composite coatings based on hydroxyapatite, chitosan, and fibroblast growth factor-2, by the MAPLE technique in order to endorse cell attachment and growth without toxic effects and to promote pre-osteoblast differentiation towards the osteogenic lineage. Our study is of interest in the context of the need for quicker bone formation and healing, which can be facilitated by various approaches such as implant coatings made from materials that favor an osseointegrating microenvironment.

## 2. Materials and Methods

### 2.1. Materials

All reagents required for the synthesis of HAp-based composite coatings were purchased from Sigma–Aldrich (Merck Group, Darmstadt, Germany), namely CaCl_2_, Na_2_HPO_4_*2H_2_O, NaOH (10%), chitosan 2% (CS) medium molecular weight, dimethyl sulfoxide (DMSO) and fibroblast growth factor 2 (FGF2).

For the biological investigations, the cell line MC3T3-E1 (ATCC^®^ CRL-2593™) was purchased from American Type Culture Collection (ATCC, Manassas, VA, USA), the fetal bovine serum (FBS) and phosphate saline buffer (PBS) from Life Technologies (Foster City, CA, USA), Alkaline Phosphatase Activity Colorimetric Assay Kit from Biovision (Milpitas, CA, USA) and the StemPro Osteogenic Differentiation Kit from Thermo Fischer Scientific (Waltham, MA, USA). All the antibodies employed for fluorescence microscopy staining were purchased from Santa-Cruz Biotechnology (Heidelberg, Germany), while the rest of the reagents and kits were purchased from Sigma-Aldrich.

### 2.2. Synthesis Methods

#### 2.2.1. Hydroxyapatite Synthesis

To synthesize the HAp powdery sample, CaCl_2_ and Na_2_HPO_4_*2H_2_O were dissolved in ultrapure water. The phosphorous-containing solution was then added dropwise to the calcium-containing solution under continuous stirring. Subsequently, the alkaline pH adjustment was performed by adding 10% NaOH, and the resulted solution underwent a one-day maturation process. The final product was subjected to filtration, triple-washing, and drying process.

#### 2.2.2. Composite Coatings Synthesis

Titanium grade 4 discs, commercially pure Ti, (with diameter and thickness of 12 mm and 0.1 mm, respectively), were used as substrates during the MAPLE experiments. Prior to surface modification by laser processing, all substrates were subjected to an ultrasonic cleaning treatment with acetone, ethanol, and deionized water (10 min each step), followed by drying under a high purity nitrogen jet.

A ns-beam (λ = 248 nm, τFWHM = 25 ns, repetition rate = 10 Hz) from an KrF* excimer COMPexPro 205 Lambda Physics from Coherent was focused on HAp/CS/FGF2 sample (irradiation spot 34 mm^2^) generating laser fluences of 200, 300, and 400 mJ/cm^2^. For MAPLE deposition, the targets were prepared from powders mixed in DMSO (2.5%) and frozen at liquid nitrogen temperature. The coatings were deposited on titanium discs and Si substrates, which were cleaned according to an internal procedure. The depositions were made for an average number of pulses of 40,000 at room temperature and 1 Pa residual gas. During the deposition, the target was continuously rotated to avoid deep crated formation, and the target to substrate distance was kept constant at 5 cm.

### 2.3. Physicochemical Investigation

#### 2.3.1. X-ray Diffraction (XRD)

The compositional identification and crystalline structure of the white powdery sample were performed using an XRD-6000 diffractometer from Shimadzu (Duisburg, Germany). The analysis was performed using the Cu_Kα_ radiation (λ = 1.056 Å) of the equipment, and the data were collected in the 20–50° range of 2θ diffraction angle.

#### 2.3.2. Transmission Electron Microscopy (TEM)

The TEM analysis of HAp powder was made with a Tecnai^TM^ G2 F30 S-TWIN high-resolution transmission electron microscope equipped with a selected area electron diffraction (SAED) accessory from FEI (Thermo Fischer Scientific, Waltham, MA, USA). The instrument operated in the transmission mode (300 kV voltage), with point and line resolutions of 2 Å and 1 Å, respectively.

#### 2.3.3. Infrared Microscopy (IRM)

The compositional analysis of HAp-based powder and coatings was performed using a Nicolet iN10 MX Fourier transform (FT)-IR microscope from Thermo Fischer Scientific. All scans were recorded in the 4000–600 cm^−1^ wavenumber range (4 cm^−1^ resolution), in the reflection mode. The IR data were processed by using the OmincPicta 8.2 software (Thermo Fischer Scientific).

#### 2.3.4. Scanning Electron Microscopy (SEM)

SEM investigation was performed on pristine HAp powder, as well as on composite coatings obtained by MAPLE. Before analysis, all samples were capped with a thin conductive gold layer. The micrographs were recorded using the secondary electron beam (30 keV) of an electronic microscope equipped with energy-dispersive X-ray spectroscopy (EDS) accessory from FEI (Thermo Fischer Scientific).

### 2.4. Biological Investigations

#### 2.4.1. In Vitro Cell Culture Model

The MC3T3-E1 cell line (CRL-2593, ATCC) was employed as the in vitro cell culture model to assess the cellular response of mouse pre-osteoblasts toward HAp/CS/FGF2 coatings, as well as for evaluating the osteogenic differentiation potential of the composites. For all biological experiments, the composites were sterilized by UV light exposure prior to cell seeding for 20 min on both sides. Non-coated substrates (titan discs) were employed as experimental control samples and were processed identically as described for the HAp/CS/FGF2-coated samples.

In order to investigate the biocompatibility of the HAp/CS/FGF2-coated samples, the MC3T3-E1 pre-osteoblasts cells were seeded at an initial density of 10^4^ cells/cm^2^ on the surface of the samples. The cellular suspension was seeded as a 20 μL drop placed onto the surface samples. After 2 h, the bioconstructs were immersed in Dulbecco’s modified Eagle medium (DMEM), supplemented with 10% FBS and 1% penicillin/streptomycin mixture (10,000 units/mL penicillin and 10 mg/mL streptomycin). The bioconstructs were maintained for 7 days under standard culture conditions (37 °C, 5% CO_2_), while refreshing the cell culture media every other day.

For the osteogenic differentiation experiments, the MC3T3-E1 pre-osteoblasts cells were seeded at an initial density of 2 × 10^4^ cells/cm^2^. The cellular suspension was seeded, and a 20 μL drop was placed onto the surface samples. The samples were left 2 h before immersion in a complete DMEM culture media to allow cellular attachment. After 24 h, the cell culture media was replaced with a commercially available osteogenic induction culture medium (StemPro Osteogenic Differentiation Kit). Under these conditions, the bioconstructs were maintained in culture for 21 days under standard culture conditions (37 °C, 5% CO_2_), while the osteogenic induction culture medium was refreshed three times a week. 

#### 2.4.2. In Vitro Biocompatibility Assessment

To investigate the biocompatibility of the HAp/CS/FGF2-coated samples, the biological response of murine pre-osteoblasts after 2 days and 7 days of interaction with the novel materials was screened by investigating cell viability, proliferation potential, and morphology, as well as measuring the cytotoxic potential of the samples.

MTT assay was employed to reveal cell viability and proliferation potential of MC3T3-E1 seeded in contact with the HAp/CS/FGF2-coated samples. Briefly, the cell culture media was discarded and replaced with 1 mg/mL 3-(4,5-dimethyldiazol-2-yl)-2,5-diphenyltetrazolium bromide MTT solution, freshly prepared in FBS-free culture media. After 4 h of incubation in the dark at 37 °C, the MTT solution was removed, and the resulting formazan crystals were dissolved in DMSO. To measure the cell metabolic health, the absorbance of the resulting solution was measured at 550 nm using a FlexStation III microplate multimodal reader (Molecular Devices, San Jose, CA, USA). To present the obtained results as a % of cell viability, the mean OD obtained for the experimental control 48 h post-seeding was considered as 100% cell viability.

To assess the cytotoxic potential of the HAp/CS/FGF2-coated samples, lactate dehydrogenase (LDH) leakage from damaged MC3T3-E1 cells was measured as a measure of cells that lost their cell membrane integrity in response to material contact. For this, cell culture media samples were harvested at the experimental time points and mixed with LDH based in vitro toxicology assay kit reagents (TOX7 kit) according to the manufacturer’s instructions. The resulting solutions were placed at room temperature in the dark for 20 min, and the reaction was stopped by adding 1 N HCl. The optic density of the solutions was finally measured by spectrophotometry at 490 nm on the FlexStation III microplate multimodal reader (Molecular Devices). To present the obtained results as % of LDH release, the mean OD obtained for the experimental control 48 h-post-seeding was considered 100% cytotoxicity.

The impact of HAp/CS/FGF2-coated samples on the morphology of MC3T3-E1 was investigated by fluorescence microscopy. After discarding the cell culture media, the HAp/CS/FGF2-coated samples were washed with PBS, immersed in a 4% paraformaldehyde solution (PFA) for 20 min for cell fixation, and further incubated for 1 h at room temperature in the 2% BSA permeabilization solution with 0.1% Triton X100. To reveal the cell morphology and nuclei, samples were subsequently stained with fluorescein isothiocyanate (FITC)-conjugated phalloidin for 1 h at 37 °C and 20 min with 4, 6-diamidino-2-phenylindole (DAPI). Microscopy micrographs were captured using the Olympus IX73 fluorescent microscope (Olympus Life Science, Waltham, MA, USA) and CellSense F software. 

#### 2.4.3. In Vitro Osteoinductive Potential Assessment

To investigate the potential of the HAp/CS/FGF2-coated samples to sustain the osteogenic differentiation process, different relevant markers for the osteogenesis process were investigated during 21-days of exposure to osteogenic inductors of MC3T3-E1 cells cultured on the HAp/CS/FGF2-coated samples, at 2 time points: 14 and 21 days.

The alkaline phosphatase (ALP) activity was determined spectrophotometrically using the Alkaline Phosphatase Activity Colorimetric Assay Kit. Briefly, the culture media was harvested at the chosen time points and mixed with the p-nitrophenyl phosphate substrate, according to the manufacturer‘s recommendations. Following the incubation step (60 min/25 °C), the optical density of the resultant p-nitrophenol (pNP) was measured at 405 nm using a FlexStation III microplate multimodal reader. To quantify the amount of pNP generated by ALP in the experimental samples, the obtained results were plotted on the p-nitrophenol standard curve. ALP activity was determined as described in the kit protocol, and the final readings were normalized against the total cell number, as recommended by the manufacturer.

To reveal the protein expression levels of the osteogenic-specific markers osteopontin (OPN) and osteocalcin (OCN), samples were fixed and permeabilized by paraformaldehyde/BSA- Triton X100 solutions as described above and incubated overnight at 4 °C, with rabbit polyclonal anti-OCN and goat polyclonal anti-OPN antibodies. Prior to fluorescence microscopy investigation, the samples were further incubated in tetramethylrodamine-5,6-isothiocyanate (TRITC)-conjugated goat anti-rabbit and FITC-conjugated rabbit anti-goat secondary antibodies solutions for 30 min at room temperature in darkness and 10 min with DAPI for nuclei staining.

The potential of HAp/CS/FGF2-coated samples to allow MC3T3-E1 cells to form calcium deposits was investigated by alizarin red S staining. Briefly, samples were washed with PBS, immersed in a 4% PFA fixative solution for 2 h, and afterward for 30 min at room temperature in a freshly prepared solution of 1% alizarin red. After staining, the dye was removed, and the samples were washed with distilled water until the washing solution remained colorless. Images were captured with the Nikon inverted optical microscope. The dye extraction was performed using a 10% acetic acid solution, and the resulting solution’s optical density was determined spectrophotometrically at 405 nm using a Flex Station III multimodal reader (Molecular Devices). Pristine HAp/CS/FGF2-coated samples were processed identically, and the data was subtracted from the obtained results to address the presence of HAp in the coating structure.

All the presented biological experiments were performed in triplicate (*n* = 3), and for the spectrophotometric assays, the results were analyzed using GraphPad 6 software (one-way ANOVA, Bonferroni test). All the data are expressed as mean ± standard error of the mean. A *p*-value of ≤0.05 was considered statistically significant.

## 3. Results and Discussions

### 3.1. Physicochemical Investigation of HAp Powder

**Figure 1:**
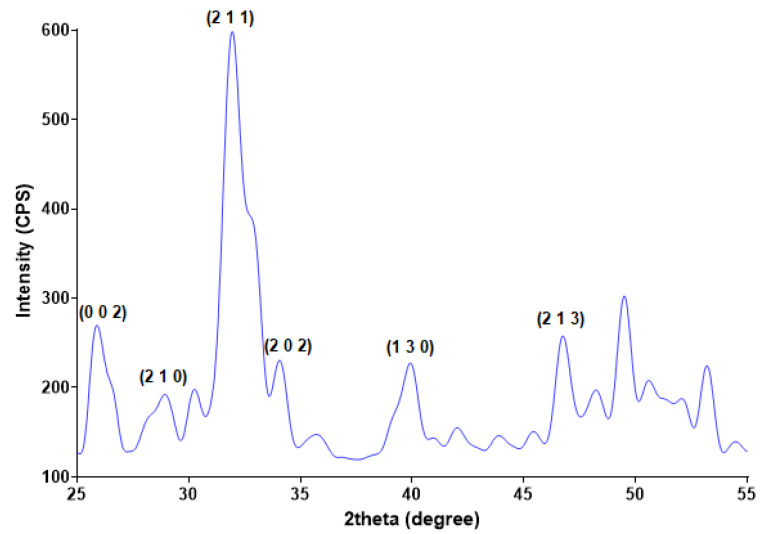
XRD pattern of HAp powder.

**Figure 2:**
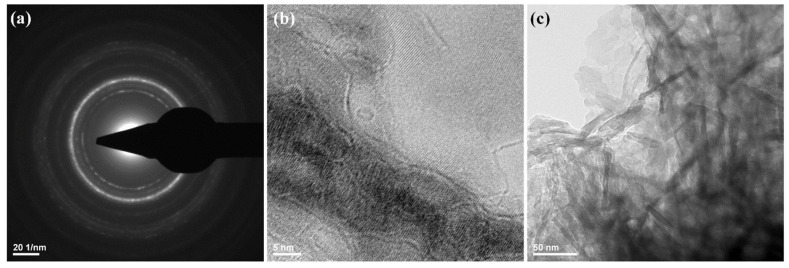
SAED pattern (**a**) and TEM images (**b**,**c**) of HAp powdery sample.

Starting from calcium and phosphorous precursors and conveniently adjusting various reaction parameters (temperature range, pH value, etc.), the hydroxyapatite (HAp) nanoparticles were obtained by the co-precipitation method [50,51]. The white powder that resulted after drying the viscous precipitate was analyzed compositional and microstructural by XRD (Figure 1) and TEM (Figure 2). The diffractogram pattern evidences the presence of broad diffraction peaks, which indicate the powder’s reduced crystallinity. Specific peaks are identified at 2θ values of 25.9°, 28.9°, 31.8°, 34°, 40°, 46.7°, and 49.5°, these maxima correspond to (0 0 2), (2 1 0), (2 1 1), (2 0 2), (1 3 0), (2 2 2), and (2 1 3) diffraction planes of hydroxyapatite crystals [52,53,54,55]. The XRD analysis confirms that hexagonal HAp represents the sole crystalline phase of the obtained powder.

The TEM measurements (Figure 2) showed the presence of HAp aggregates, consisting of needle-like nanosized individual particles. Those observations were in good agreement with other reports on the microstructure of synthetic HAp [56,57,58]. The HAp obtained has a width of ~10 nm and a length between 10−80 nm.

Structural information about HAp nanoparticles was also obtained from SAED analysis, as presented in Figure 2a. The identified diffraction rings indicate the polycrystalline nature of prepared nanostructures. The SAED pattern confirms the synthesis of a high purity HAp sample.

### 3.2. Physicochemical Characterization of HAp/CS/FGF2 Coatings

**Figure 3:**
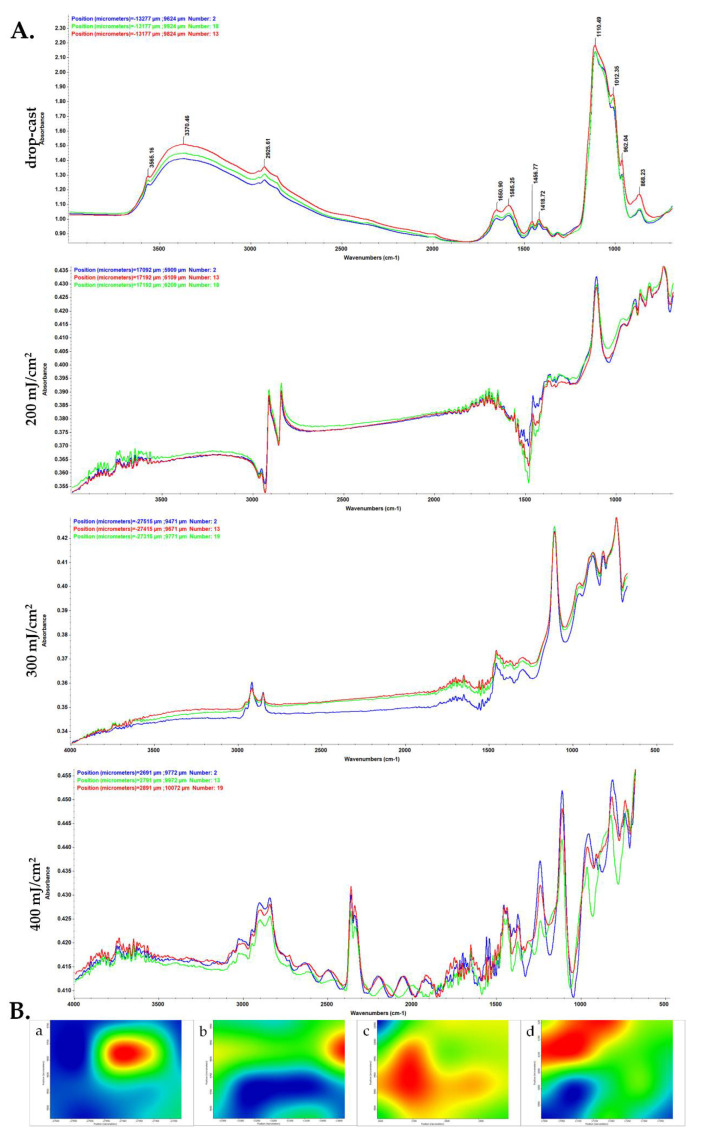
IR micrographs (**B**) and corresponding IR spectra (**A**) of HAp/CS/FGF2 coatings at (**b**) 200, (**c**) 300 and (**d**) 400 mJ/cm^2^ laser fluences and (**a**) drop-cast.

In order to investigate the compositional integrity of the composite laser-processed materials, comparative infrared studies were conducted between the drop-cast sample and the coatings obtained by MAPLE (Figure 3). Fourier transform infrared spectroscopic (FT-IR) analysis mainly provides information about the functional properties which correlate with functional group and structure of composite blended coatings.

The FT-IR spectral details of chitosan showed that the OH peaks can be assigned at ~3565 cm^−1^, and alkyl C–H stretching vibration was identified at ~2925 cm^−1^ [59]. Strong peaks were observed at ~1650 cm^−1^, ~1585 cm^−1^ and ~1456 cm^−1^ showing the presence of C=O stretching (amide-I band) [60], N-H bending, and C-H deformation, respectively. In the IR spectra of initial material (drop-cast), absorption maxima that originate from HAp can be identified, such as stretching vibrations from structural hydroxyl groups (~3565 cm^−1^), ν3 asymmetric stretching of PO_4_^3−^ (~1110, ~1012 cm^−1^) and PO_4_^3−^ ν1 stretching (~962 and ~868 cm^−1^) [61].

When compared to the drop-cast, the lowest laser fluence (200 mJ/cm^2^) did not affect the chemical integrity of HAp/CS/FGF2 material. Slightly modified and reduced IR maxima indicate an insufficient amount of transferred material, as confirmed by the presence of predominant blue areas in the complimentary infrared maps. An increased transfer of HAp/CS/FGF2 is noticed for the 400 mJ/cm^2^ fluence. In terms of efficient and uniform transfer of HAp/CS/FGF2 material and preserved chemical integrity, optimal results are evidenced by using the 300 mJ/cm^2^ laser fluence. As a general remark regarding the IR microscopy analysis, the absorbance intensity of the collected infrared spectra is directly related to the color changes within the resulted IR maps, ranging from blue to red (corresponding to the lowest and highest intensity, respectively).

The absorbance maxima previously identified for synthetic HAp can also be noticed in the IR spectra of drop-cast and MAPLE processed HAp/CS/FGF2 materials. The infrared peaks identified at ~2925 cm^−1^ (which may result from overlapped stretching of hydroxyl from HAp and N–H from primary amines of organic compounds), ~1110 cm^−1^, 868 cm^−1^ (PO_4_^3−^ stretching), and ~1418 cm^−1^ (stretching and bending vibrations of carbonaceous bonds from organic molecules), confirm the successful transfer of HAp/KAN/FGF2 composite materials.

**Figure 4:**
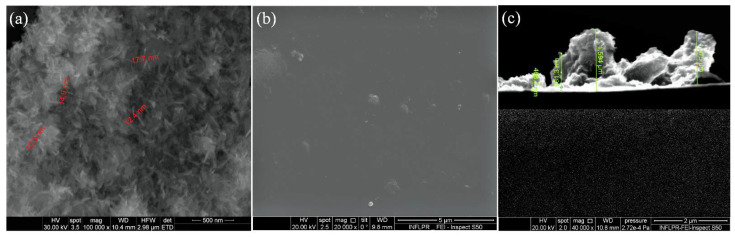
SEM images of HAp/CS/FGF2 (**a**,**b**) coatings obtained at 300 mJ/cm^2^ laser fluence and (**c**) cross section.

Relevant microstructural data about the obtained HAp/CS/FGF2 coatings were collected from the SEM analysis, corresponding images are presented in Figure 4 and offer information regarding the textured aspect of the coating formed at a laser fluence of 300 mJ/cm^2^ (Figure 4a,b). Figure 4c provides useful data concerning the thickness of the nanostructured coatings obtained at 300 mJ/cm^2^ laser fluence. As it can be seen, the thickness varies between 400 nm and 1 µm, which delineates the small agglomeration tendency.

### 3.3. Biocompatibility Evaluation of HAp-Based Coatings

To investigate whether the HAp/CS/FGF2 coatings fulfill the basic requirements of a biocompatible material, MC3T3-E1 cell adhesion, viability, and proliferation, as well as the cytotoxic potential of the samples were assessed.

**Figure 5:**
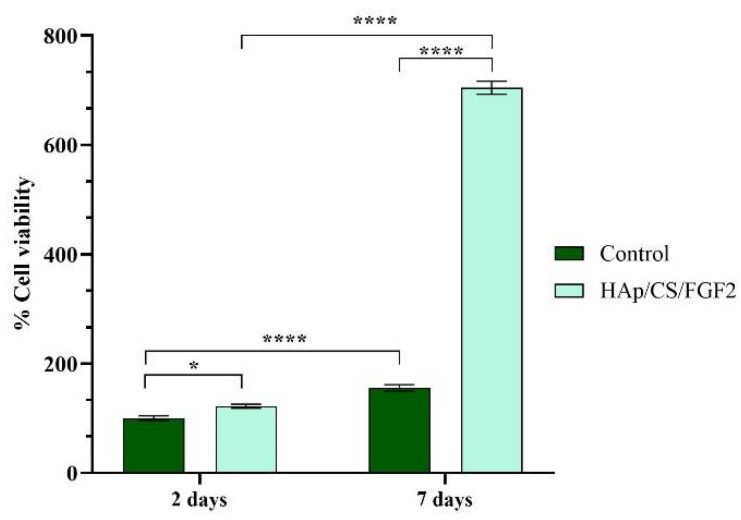
Graphical representation of MC3T3-E1 cell viability and proliferation potential after 2 days and 7 days of contact with control and HAp/CS/FGF2 samples (* *p* < 0.05; **** *p* < 0.0001).

The cellular metabolic activity revealed by the spectrophotometric MTT assay (Figure 5) showed a significant increase (*p* < 0.05) of viable MC3T3-E1 cells cultured on HAp/CS/FGF2 coatings as compared with control samples even after 2 days of culture. While on both samples, the cell viability increased gradually with the prolonged culture time, the coating strategy significantly increased (*p* < 0.0001) the number of viable cells adhered on the material surface in comparison with the control sample after 7 days of culture. Moreover, both samples sustained and promoted MC3T3-E1 cell proliferation as revealed by the significant increase (p < 0.0001) of cell viability 7 days post-seeding compared to 2 days. However, on HAp/CS/FGF2-coated samples, a significant enhancement of cell proliferation was observed, showing that surface tuning stimulates pre-osteoblast proliferation. This dramatic increase of cell proliferation on HAp/CS/FGF2 could be attributed to the presence of FGF2, which is a well-known molecule involved in stimulating osteoblasts proliferation [62,63].

**Figure 6:**
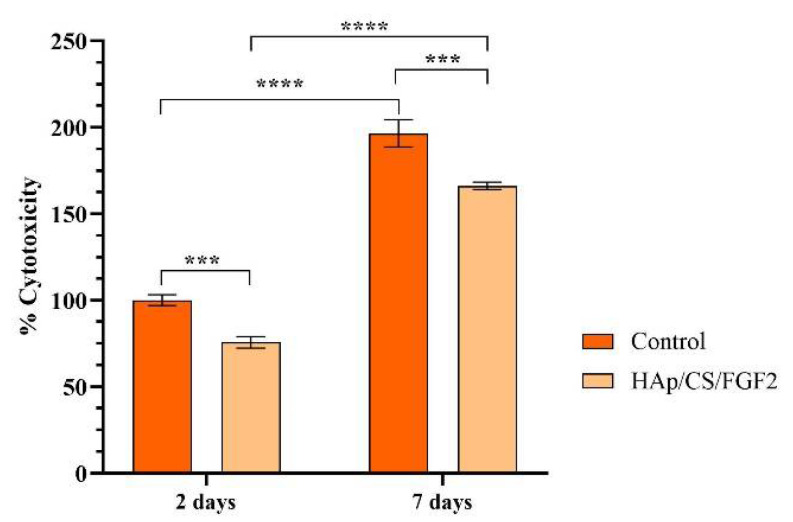
Graphical representation of LDH leakage levels from damaged MC3T3-E1 cells after 2 days and 7 days of contact with control and HAp/CS/FGF2 samples as a measure of materials cytotoxicity (*** *p* < 0.001; **** *p* < 0.0001).

To reveal the cytotoxic potential of the samples, the amount of LDH released in the culture media by damaged MC3T3-E1 cells that possess compromised cell membranes in response to materials contact was measured (Figure 6). After 2 days of culture, the LDH levels in the culture media collected from the HAp-based samples were significantly lower (*p* < 0.001) as compared with the control sample. The same pattern of the LDH release was also observed after 7 days of culture, highlighting that the coating absence induces a significant higher (*p* < 0.001) damage on pre-osteoblast cells compared with the HAp/CS/FGF2-coated samples that exhibited low cytotoxicity.

**Figure 7:**
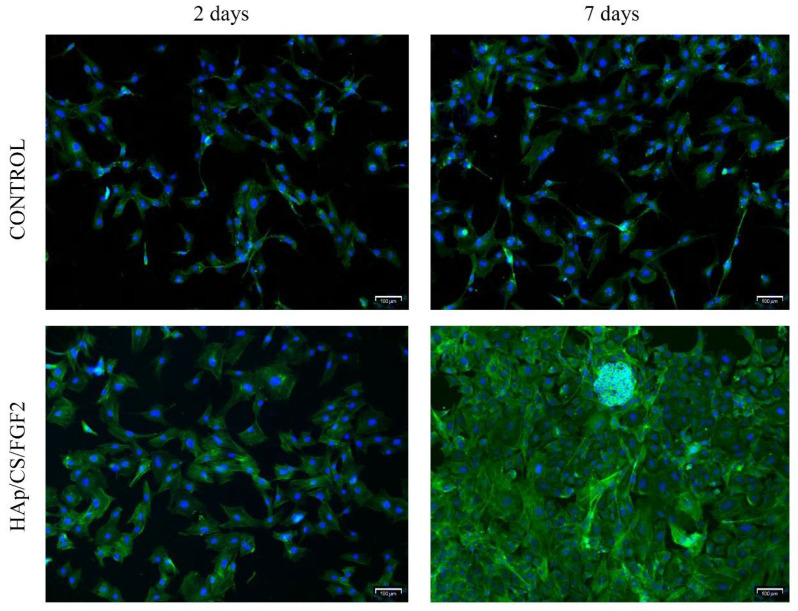
Fluorescence microscopy micrographs (scale bar 100 μm) highlighting the cytoskeleton of MC3T3-E1 cells 2 days and 7 days post-seeding on control and Hap/CS/FGF2 samples. Actin filaments were stained with phalloidin-FITC (green) and cell nuclei with DAPI (blue).

Fluorescence microscopy was employed to study MC3T3-E1 cell behavior and morphology after HAp/CS/FGF2-coated samples interaction (Figure 7). After 2 days of culture, cells were capable of adhering to both samples, no differences being noticed in the cell distribution pattern on the sample’s surfaces or cell morphology. However, pre-osteoblasts responded slightly differently to HAp/CS/FGF2 coatings as a larger number of cells were noticed on the material surface, which presented a more well-developed cytoskeleton, most probably because the coating favored a more tight cell adhesion. After 7 days of culture, in the control sample, no differences were observed in the cell morphology compared with 2 days, the pre-osteoblasts still expressing low levels of actin, condensed around the nuclei and lacking organization, the sample surface being randomly scattered with cells. On the other hand, on the HAp/CS/FGF2-coated samples, pre-osteoblasts were organized in a compact cellular network distributed evenly on the entire material surface. Probably because the entire surface was covered with cells and also, as revealed by the MTT assay, the coating strategy highly stimulated cellular proliferation on different parts of the material, cells organized in 3D cellular structures.

The biocompatibility screening revealed that the coating strategy significantly improves the biological performance of the metallic implants as the HAp/CS/FGF2 coating sustains cell viability and proliferation, and overall sustains MC3T3-E1 cell development. This positive outcome is determined by the chosen materials for the enrichment of the metallic implants. On one hand, HAp is a major component of the bone and is widely used for bone tissue engineering biomaterials fabrication due to its excellent bioactivity and osteoconductivity [64]. On the other hand, CS also lacks toxicity and is highly biocompatible, but due to poor mechanical properties and lack of osteoconductivity, it cannot be used solely for bone tissue engineering purposes [65]. However, CS supports osteoblasts’ adhesion and proliferation, as well as in vitro formation of mineralized bone matrix in vitro [66], and therefore, HAp is an ideal candidate to improve the mechanical properties of CS. Shahzadi et al. showed an enhancement of cell adhesion, spreading, and proliferation of MC3T3-E1 cells on Nylon 6/CS/HAp nanofibers compared with pristine Nylon 6 fibers showing that by tunning the material with HAp and CS, a better environment for cellular development is achieved [67]. Excellent cytocompatibility with murine and human pre-osteoblasts was also observed in response to interaction with a CS/agarose/Hap scaffold [68]. Moreover, Hap/CS composites induce and sustain osteoinduction and in vivo studies showed that Hap/CS composites promote bone formation in different bone defects [69,70].

### 3.4. Osteoinductive Potential Evaluation of HAp-Based Coatings

To assess if the HAp/CS/FGF2 coatings favor and sustain the osteogenic differentiation of MC3T3-E1 pre-osteoblasts towards the osteogenic lineage, cells were exposed to osteogenic inductors for 21 days, during which time various characteristics of the early and late stages of this process were screened.

**Figure 8:**
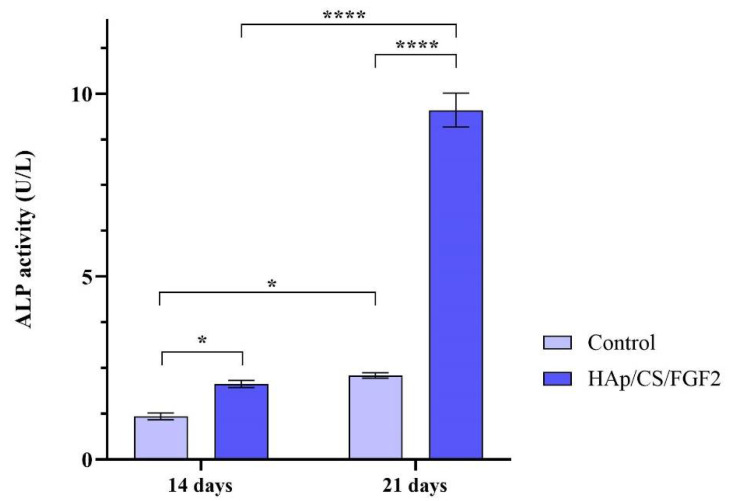
ALP activity assessment in culture media harvested from MC3T3-E1 cells seeded on control samples and HAp/CS/FGF2 coatings during 21 days of exposure to osteogenic inductors (* *p* < 0.5; **** *p* < 0.0001).

ALP activity measurement was performed after 14 and 21 days of culture to monitor the osteogenic differentiation process in the early phase as the enzyme activity increases, starting with the early stages of osteoblasts’ commitment [71]. The obtained results (Figure 8) showed that both experimental samples increased the activity of ALP over time, but a significantly higher ALP enzyme activity was observed in the media samples collected from HAp/CS/FGF2-coated samples as compared with the control samples after 14 (*p* < 0.05) and 21 days of culture (*p* < 0.0001). While in the non-coated samples, a modest increase of the ALP activity was obtained after 21 days of culture as compared with 14 days of culture, the HAp/CS/FGF2-coated samples significantly enhanced the enzyme activity. After 3 weeks of culture, the ALP activity in the HAp/CS/FGF2-coated samples significantly increased (*p* < 0.0001) compared to 2 weeks of culture. This 4.6-fold increment of the ALP activity between the 2 time points highlights that in contact with HAp/CS/FGF2-coated, more and more pre-osteoblasts developed into mature osteoblasts.

**Figure 9:**
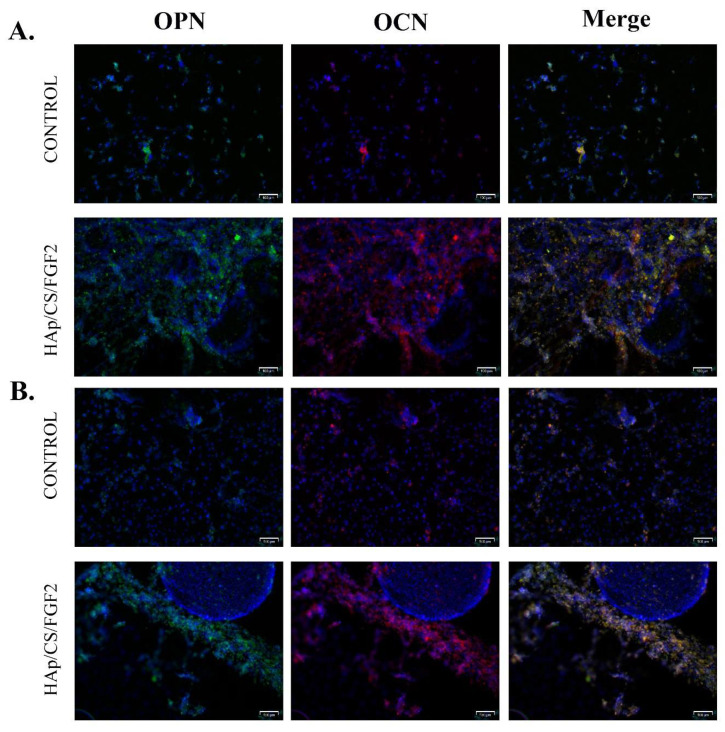
Fluorescence micrographs (scale bar 100 μm) revealing the protein expression of osteogenic specific markers OPN (green) and OCN (red) after 14 (**A**) and 21 (**B**) days of MC3T3-E1 cells’ exposure to osteogenic inductors in contact with control surfaces and HAp/CS/FGF2 coatings. Cell nuclei are stained with DAPI (blue).

Furthermore, the protein expression of osteogenesis-related markers OPN and OCN was investigated by fluorescence microscopy, both proteins being produced by differentiated osteoblasts in the late stages of the process (Figure 9). The immunofluorescence staining to reveal the protein expression of OPN and OCN was carried out on day 14 and day 21 post-culture initiation and differentiation induction. After 14 days of osteogenic induction of MC3T3E1 cells, the positive expression of OPN and OCN was observed in both non-coated and HAp/CS/FGF2-coated samples. However, a significantly lower ratio of cells expressing OPN and OCN was observed in control samples. In contrast, the HAp/CS/FGF2-coating stimulated the expression of OPN and OCN, with almost all cells on the material surface expressing OPN and OCN proteins at different levels. In contact with the HAp/CS/FGF2 coatings, cells organized tightly in a 3D cellular network, the expression of both OPN and OCN being lower where cells formed packed cell clusters. After 21 days of osteogenic induction, no increase in the OPN and OCN protein expression was triggered by cell contact with the reference samples. On the HAp-based coating samples, cells were organized into a more evolved 3D tubular-like structure, where all cells still expressed OPN and OCN. Moreover, a small population of cells detached from the 3D compact cellular network formed on HAp/CS/FGF2 samples and condensate into a 3D cellular spheroidal structure, where the expression of OPN and OCN were not identified. At this level, most probably, cells that generated this 3D cellular spheroidal structure transitioned to the condensation phase and, as a result, lost their active state in which they are able to synthesize these proteins.

**Figure 10:**
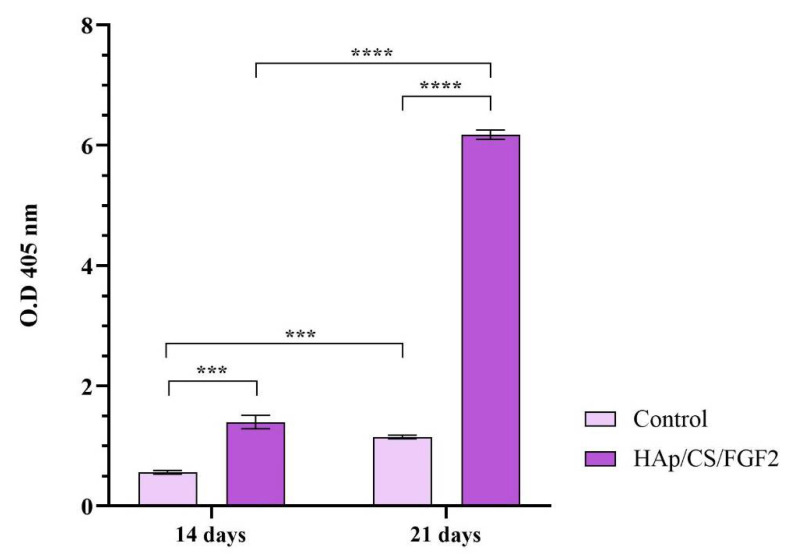
Quantification of the alizarin red S staining after 14 and 21 days of MC3T3-E1 osteogenic differentiation in contact with control samples and HAp/CS/FGF2 coatings (*** *p* < 0.001; **** *p* < 0.0001).

To monitor the calcification process of MCT3-E1 cells cultured in contact with non-coated and HAp/CS/FGF2 coatings and exposed to pro-osteogenic stimuli, alizarin red S staining was employed (Figure 10). After 14 days of culture, the positive presence of calcium deposits was identified in both non-coated and coated samples, with a significant increase (*p* < 0.001) of the mineralization process in HAp/CS/FGF2 coatings samples. After 21 days of culture, a significant acceleration of the calcification process was noticed in control and HAp-based coating samples, sustained by the increase of the number of calcium deposits as compared with levels registered at 14 days of culture. Even though both substrates promoted at this time point to the calcification process, a significant enhancement (*p* < 0.001) of the mineralization process was triggered by cell contact with HAp/CS/FGF2 coatings as compared with non-coated samples, as highlighted by the 4.4-fold increase of the positive cells for alizarin red S staining that show that surface coating promotes the extracellular matrix mineralization process.

The obtained results show that the novel HAp/CS/FGF2 favors the osteogenic differentiation of MC3T3-E1 pre-osteoblasts in comparison with the reference sample, where a modest differentiation process was observed. These significant changes are most probably due to the coating composition, the HAp, and CS facilitating both early and late stages of the differentiation process by stimulating the ALP activity, and enhancing the mineralization process [72,73].

## 4. Conclusions

Suitable coatings were produced for bone tissue engineering applications that lack toxicity and promote cell adhesion and proliferation while sustaining the differentiation of pre-osteoblasts towards mature bone cells. The obtained results showed that the biocompatibility of the metallic implants is significantly increased by the HAp/CS/FGF2 coating, as highlighted by the superior potential in favoring cell adhesion and sustaining cell viability and proliferation, exhibiting low cytotoxicity as compared with uncoated samples. This profile was attributed to the coating’s structure, which was designed based on HAp and CS, both excellent candidates for biomedical applications precisely for their low capacity to induce cell toxicity. Proliferative status enhancement of the pre-osteoblasts cells was attributed to the FGF2 blended in the coating status; this growth factor is associated with cell proliferation stimulation. The potential of the HAp/CS/FGF2 coatings to stimulate osteogenic differentiation of MC3T3-E1 cells exposed to osteogenic inductive conditions was assessed. Different scenarios of the osteogenic differentiation process were screened and revealed that the HAp/CS/FGF2 coatings endorse the metallic implant with osteoinductive properties. Cells in contact with the HAp/CS/FGF2-coated sample showed significantly elevated ALP activity, enhanced in vitro mineralization process, and expression of osteogenic-related key markers OPN and OCN. Therefore, independent of the osteogenic inductors supplied continuously, pre-osteoblasts fail to differentiate in mature bone cells when lacking the appropriate substrate. The reported results indicate that the HAp/CS/FGF2 coatings are a suitable approach for improving traditional titanium metallic implants. This coated-material could be further employed to guide and enhance bone regeneration.

## Data Availability

Not applicable.
